# Bone marrow-derived mesenchymal stem cells attenuate myocardial ischemia–reperfusion injury via upregulation of splenic regulatory T cells

**DOI:** 10.1186/s12872-021-02007-4

**Published:** 2021-04-27

**Authors:** Ling-Xiao Pang, Wen-Wei Cai, Qian Li, Heng-Jie Li, Min Fei, Yong-Sheng Yuan, Bin Sheng, Ke Zhang, Rong-Cheng An, Ying-Wei Ou, Wen-Jie Zeng

**Affiliations:** 1 Department of Emergency, Zhejiang Provincial People’s Hospital,People’s Hospital of Hangzhou Medical College, Hangzhou, 310014 China; 2 Department of Health Management Center, Zhejiang Provincial People’s Hospital,People’s Hospital of Hangzhou Medical College, Hangzhou, 310014 China; 3 Department of Gynecology, Zhejiang Provincial People’s Hospital, People’s Hospital of Hangzhou Medical College, ShangTang Road 158, Hangzhou, 310014 China

**Keywords:** Bone marrow-mesenchymal stem cells, T regulatory cells, Ischemia–reperfusion injury, Spleen, Interleukin 10, Transforming growth factor β1

## Abstract

**Background:**

Myocardial ischemia–reperfusion injury (MIRI) is the main pathological manifestation of cardiovascular diseases such as myocardial infarction. The potential therapeutic effects of bone marrow-derived mesenchymal stem cells (BM-MSCs) and the participation of regulatory T cells (Tregs) in MIRI remains to be defined.

**Methods:**

We used the experimental acute MIRI that was induced in mice by left ascending coronary ischemia, which were subsequently randomized to receive immunoglobulin G (IgG) or anti-CD25 antibody PC61 with or without intravenously injected BM-MSCs. The splenectomized mice underwent prior to experimental MIRI followed by intravenous administration of BM-MSCs. At 72 h post-MIRI, the hearts and spleens were harvested and subjected to cytometric and histologic analyses.

**Results:**

CD25^+^Foxp3^+^ regulatory T cells were significantly elevated after MIRI in the hearts and spleens of mice receiving IgG + BM-MSCs and PC61 + BM-MSCs compared to the respective control mice (all p < 0.01). This was accompanied by upregulation of interleukin 10 and transforming growth factor β1 and downregulation of creatinine kinase and lactate dehydrogenase in the serum. The post-MIRI mice receiving BM-MSCs showed attenuated inflammation and cellular apoptosis in the heart. Meanwhile, splenectomy compromised all therapeutic effects of BM-MSCs.

**Conclusion:**

Administration of BM-MSCs effectively alleviates MIRI in mice through inducing Treg activation, particularly in the spleen.

**Supplementary Information:**

The online version contains supplementary material available at 10.1186/s12872-021-02007-4.

## Backgrounds

Although the incidence of myocardial infarction (MI) has decreased in recent years, it remains a major global threat to human health and healthcare costs (1). Mortality from MI has been reduced, however, through the development and use of reperfusion therapy such as pharmacological thrombolytic agents, coronary artery bypass surgeries, and percutaneous coronary intervention (2). Inadvertently, further myocardial damage may occur as a consequence of blood flow restoration, resulting in complications that may affect cardiac function long-term. This phenomenon was termed myocardial ischemia–reperfusion injury (MIRI) by Jennings et al. in 1960 (3). It was later demonstrated that microstructural, functional, metabolic, and electrophysiological alterations in the myocardium also may occur as part of post-reperfusion tissue damage, and present as clinical manifestations of arrhythmia, myocardial stunning, no-reflow, and sometimes even death (< superscript citation needed here) (4).In that preclinical animal model of acute MI, the extent of MIRI is shown to be as high as 50% of the total infarction area in fatal MIRI. Thus, preventive and therapeutic approaches for MIRI have been greatly sought.

Previous studies have demonstrated that regulatory T cells participate in myocardial ischemia–reperfusion injury and play an important role in tissue repair (5–7). The homeostasis of the T cell population was found to be altered after experimentally induced MIRI in mice, and the adoptive transfer of exogenous Tregs attenuated atrial remodeling (6). The PI3K/Akt pathway is critical in the induction of Tregs by N,N-dimethylsphingosine in mice subjected to experimental MIRI by left main coronary artery occlusion (5). In a similar MIRI animal model, activated Tregs exerted their anti-apoptotic properties in a CD39-dependent manner (7). Presently, the therapeutic potential of bone marrow-derived mesenchymal stem cells (BM-MSCs) has been demonstrated in preclinical MIRI studies and shown to enhance left ventricular ejection fraction, myocardial blood flow, and atrial reconstruction (8–10). BM-MSCs have also been shown to exhibit immunomodulatory and anti-inflammatory functions (11–13), where their production of various cytokines, including interleukin 10 (IL-10) and transforming growth factor β1 (TGF-β1), promotes numbers of regulatory T cell (Treg) (12, 13). Furthermore, MSCs rely on a successful splenocyte activity in order to induce Tregs that suppress ischemic-induced tissue inflammation and injury, and splenectomy prevents such a phenomenon (14). To date, the immunomodulatory and Treg promoting capacities of BM-MSCs have not been explored in the context of concurrent MIRI. The present study aimed to examine changes in the Treg population after administration of BM-MSCs in a murine MIRI model, and to evaluate the roles of Tregs and spleen in the amelioration of MIRI after post-BM-MSCs therapy.

## Methods

### Mouse MIRI model

Left anterior ascending coronary artery (LAD) ligation was performed in male C57BL/6 mice weighting 20–25 g prior to surgery to induce acute MIRI according to a protocol published previously (15). In brief, mice were anesthetized by chloral hydrate (10% chloral hydrate, 0.3 ml/100 g mouse weight) administered peritoneally, secured onto the operating surface, and intubated in the supine position. Mice were then connected to a small rodent ventilator and an electrocardiogram throughout the experiment. After exposing the heart by cutting through the intercostal space between the 3^rd^ and 4^th^ sternal ribs, LAD was temporary blocked by a ligation tightened around a medical latex tube (socket inner diameter, 1.5 mm) placed between the ligature and the LAD to induce myocardial ischemia. After 30 min of successful ischemia confirmed by S-T segment elevation in inferior lead II, the suture and latex tube were withdrawn to reperfuse the myocardium. For mice receiving splenectomy, the spleen was removed according to a previously published protocol (16) hours prior to LAD ligation. The euthanasia procedure was performed according to NIH guideline for euthanasia of rodents using carbon dioxide states that a CO2 fill rate of 30- 70% of the chamber volume per minute is to be used when euthanizing rodents. All experimental procedures related to animals were performed in accordance with the Guide for the Care and Use of Laboratory Animals, performed according to ARRIVE guidelines. Experimental facilities and laboratory space was kindly provided by Hangzhou Hibio Bio-tech Co., Ltd. (Hangzhou, China). The ethical approval was obtained from Hangzhou Hibio Animal Care and Use Committee (IACUC NO. HB2019011904001PXX-A).

### Post-MIRI treatments

Six hours after the reperfusion, mice were randomly selected into separate groups to receive saline or commercially developed RFP-labeled BM-MSCs (1 × 10^6^ cells per mouse; Cyagen, Suzhou, China) in combination with Treg-depleting anti-CD25 PC61 monoclonal antibody or control immunoglobulin G (IgG) (both antibodies at 200 μg per mouse; Humanwell Biocell Biotechnology Co., Ltd, Zhengzhou, China) via the tail vein.

### Tissue collection

Seventy-two hours after MIRI induction, mice were euthanized and their target tissues and whole blood collected. A portion of the heart tissue was saved and processed for histological examination and MI area measurements. Cells were isolated from freshly harvested heart and spleen tissues. For this purpose, tissues were rinsed twice with pre-chilled phosphate buffered saline (PBS), and immersed in pre-chilled RPMI 1640 medium. Cells were subsequently dissociated from tissue samples by mincing and passing through a mesh strainer. Red blood cells (RBC) in the tissue cell suspension were lysed by incubating with RBC lysis buffer (MultiSciences Biotech, Co., Ltd, Hangzhou, China) followed by appropriate washes with PBS. Serum was collected after the blood samples had clotted and stored appropriately for target protein quantification at a later date.

### Identification of Tregs

Cell suspensions freshly prepared from the spleen and the heart were triple-stained with anti-CD4-FITC, anti-CD25-APC, and anti-Foxp3-PE antibodies following the instructions and concentration recommended by the manufacturer (Invitrogen eBioscience, catalog No. 88–8111-40). For the identification of Tregs on flow cytometry, a CD4 positive gate was first applied and followed by a CD25-Foxp3 double-positive gate.

### ELISA analysis

The activity of serum creatine kinase (CK) and lactate dehydrogenase (LDH), which are biomarkers of myocardial injury, were assessed using commercial biochemical kits (Nanjing Jiancheng Bioengineering Institute, Nanjing, China) according to the manufacturer’s instructions. The concentrations of serum IL-10 and TGF-β1 were measured by commercial ELISA kits according to the user’s manual (MultiSciences Biotech, Co., Ltd, Hangzhou, China).

### H&E staining

Formalin-fixed, paraffin-embedded 4 μm sections of heart tissues were stained with hematoxylin and eosin (HE; Sigma-Aldrich, St. Louis, MO, USA) and examined by light microscopy.

### TUNEL assay

After an appropriate pretreatment of heart tissue sections with proteinase K, the extent of cellular apoptosis in the myocardium was examined using a commercial TUNEL assay (Roche, Basel, Switzerland) following the package insert. Apoptotic cells were counted manually in five non-overlapping fields randomly selected from each tissue section at 200 × magnification.

### MI area measurements

The harvested heart tissues were sliced and immersed in 2% 2,3,5-triphenyltetrazolium (TTC; Sigma-Aldrich, St. Louis, MO, USA) solution protected from light. After 15–30 min, the tissue slices were transferred and fixed in 4% acetone for 24 h. The MI area is expressed as the averaged percent of total ventricular area.

### Statistical analysis

All data are expressed as mean ± standard deviation (SD). The differences between more than 2 groups were analyzed by one-way ANOVA, while those between 2 groups were analyzed by the least significant difference (LSD) test. P values < 0.05 were considered statistically significant. All statistical analyses were performed by SPSS version 19.0 (IBM, Armonk, NY, USA).

## Results

### BM-MSCs administration increases numbers of Treg cells in MIRI mice

**Fig. 1:**
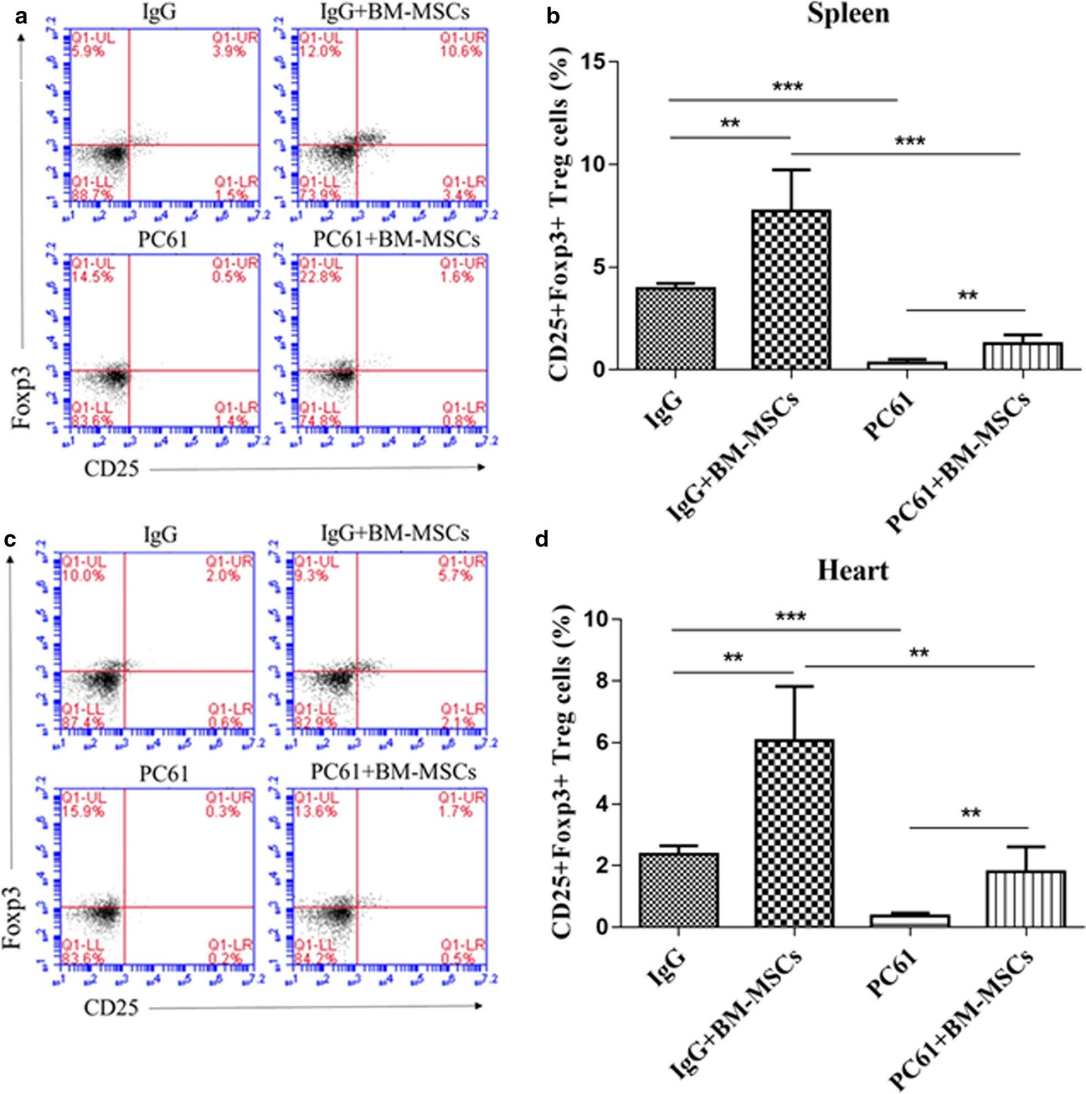
CD25^+^Foxp3^+^Tregs in the heart and spleen were increased by BM-MSCs administration in MIRI mice. Representative flow cytometry scatter plots confirm CD25 + Foxp3 + Tregs in spleen (**a**) and heart (**b**) from mice treated with IgG, IgG + BM-MSCs, PC61, and PC61 + BM-MSCs (n = 5 in each group) at indicated time. The group quantitation results of CD4 + CD25^+^Foxp3^+^ Tregs in the spleen (**c**) and the heart (**d**) are shown. Tissues were collected 72 h after surgically-induced MIRI. The bars shown in C and D represent the mean value ± standard deviation of each indicated group. BM-MSCs, bone marrow-derived mesenchymal stem cells; MIRI, myocardial ischemia–reperfusion injury; Treg, regulatory T cells. SEM ± SD, *p < 0.05, **p < 0.01 and ***p < 0.001

After treatment with IgG + BM-MSCs, the number of CD25^+^Foxp3^+^ Tregs were significantly increased by almost 2- to three-fold compared with treatment with IgG alone in both the heart and spleen of mice with 72 h post-MIRI (IgG *vs* IgG + BM-MSCs, p < 0.01) (Fig. 1). Depletion of Tregs by PC61 treatments was observed in both mice receiving antibody alone and in combination with BM-MSCs 72 h after MIRI induction (IgG *vs* PC61 and IgG + BM-MSCs *vs* PC61 + BM-MSCs, both p < 0.001) (Fig. 1). Of note, the cardiac and splenic Treg pool depleted by PC61 was significantly replenished by BM-MSCs (PC61 *vs.* PC61 + BM-MSCs, p < 0.01), so that it was numerically comparable to that by IgG alone (IgG *vs* PC61 + BM-MSCs, p > 0.05) (Fig. 1).

### BM-MSCs treatment increased the serum concentration of immunomodulatory cytokines in MIRI mice

**Fig. 2:**
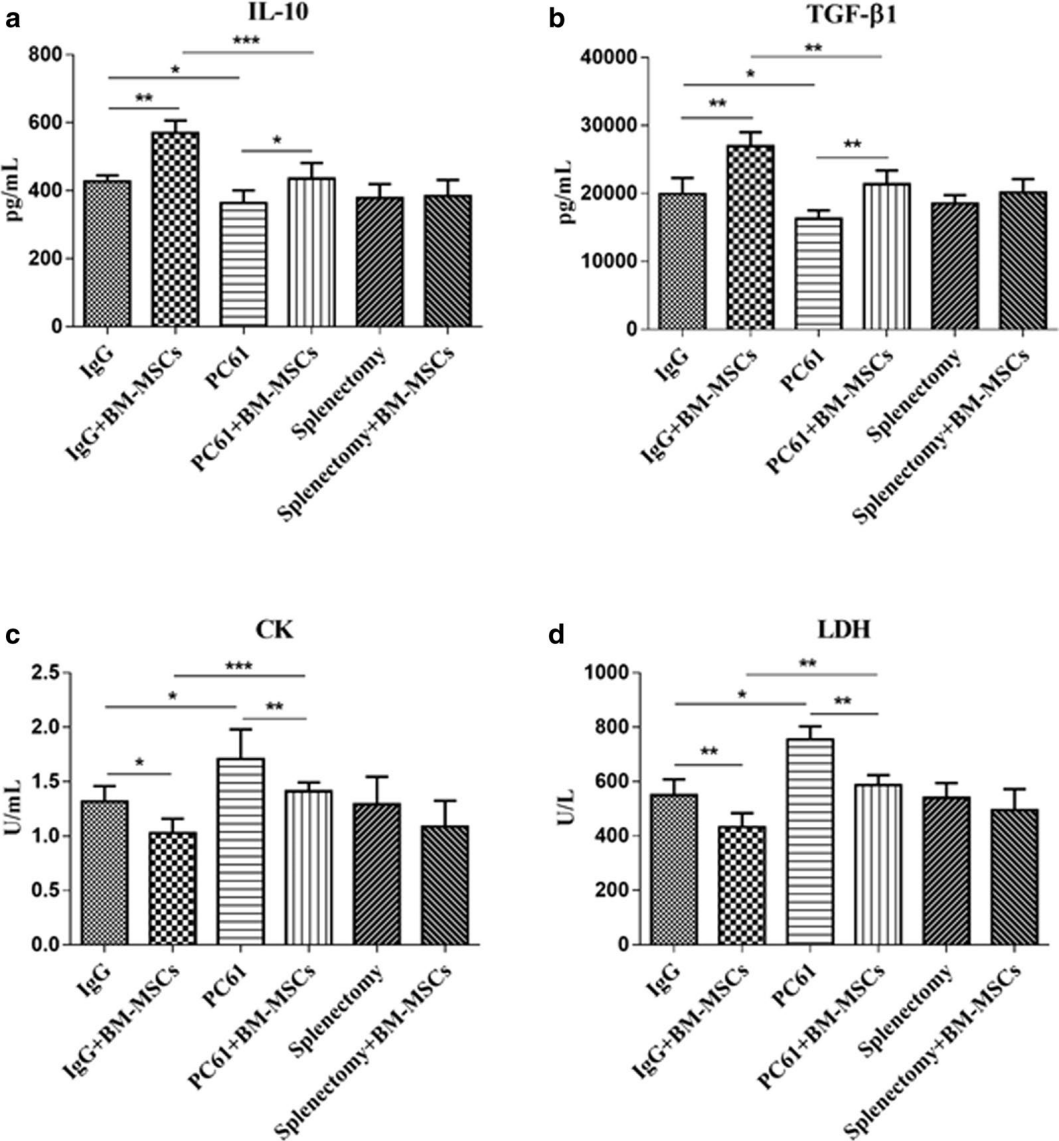
BM-MSCs treatment increased the level of IL-10 and TGF-β1 cytokines and decreased the level of CK and LDH in the serum of MIRI mice. The level of IL-10 (**a**), TGF-β1 (**b**), CK (**c**), and LDH (**d**) in the serum of mice treated with IgG, IgG + BM-MSCs, PC61, PC61 + BM-MSCs, splenectomy, and splenectomy + BM-MSCs (n = 5 in each group) are shown. Whole blood was collected 72 h after surgically-induced MIRI, from which the serum was separated. IL-10 and TGF-β1 were measured by ELISA, while CK and LDH were measured by enzyme assays. The bars represent the mean value ± standard deviation of target proteins in each indicated group. BM-MSCs, bone marrow-derived mesenchymal stem cells; CK, creatinine; LDH, lactate dehydrogenase; MIRI, myocardial ischemia–reperfusion injury. SEM ± SD, *p < 0.05, **p < 0.01 and ***p < 0.001

The serum concentrations of IL-10 and TGF-β1 were significantly higher in the group receiving IgG + BM-MSCs compared to those receiving IgG alone 72 h post-experimentally induced MIRI (IgG *vs* IgG + BM-MSCs, both cytokines p < 0.01; Table 1 and Fig. 2a, b). Mice receiving PC61 alone to deplete Tregs had significantly lower levels of the two immunomodulatory cytokines compared with those receiving IgG alone. (IgG *vs* PC61, both cytokines p < 0.05 Table 1 and Fig. 2a, b). In contrast, administration of BM-MSCs to the mice to replenish the Treg pool depleted by PC61 showed a concurrent and significant increase in the serum level of IL-10 and TGF-β1 (Table 1 and PC61 *vs.* PC61 + BM-MSCs, both p < 0.01; Fig. 2a, b) to a similar concentration observed in mice that received isotype and saline control (Table 1 and IgG *vs* PC61 + BM-MSCs, both cytokines p > 0.05; Fig. 2a, b).

**Table 1:** The serum level of IL-10, TGF-β1, CK, and LDH 72 h after MIRI

Group	IgG	IgG + BM-MSCs	PC61	PC61 + BM-MSCs	Splenectomy	Splenectomy + BM-MSCs
IL-10 (pg/mL)	426.7 ± 18.5	569.87 ± 36.25	363.71 ± 37.44	434.79 ± 46.68	378.1 ± 41.8	384.32 ± 47.18
TGF-β1 (pg/mL)	19,901.1 ± 2387.9	26,955.38 ± 2077.85	16,287.67 ± 1220.69	21,352.49 ± 2018.52	18,501.3 ± 1257.9	20,137.69 ± 1965.71
CK (U/mL)	1.3 ± 0.1	1.03 ± 0.13	1.71 ± 0.27	1.41 ± 0.08	1.29 ± 0.25	1.08 ± 0.24
LDH (U/L)	549.8 ± 58.0	433.09 ± 50.72	754.44 ± 48.51	587.17 ± 36.54	540.4 ± 54.0	495.48 ± 76.70

### BM-MSCs treatment reduced the level of MIRI-induced biomarkers in the serum

After treatment of BM-MSCs, the serum levels of CK and LDH induced by MIRI was significantly reduced (Table 1 and IgG *vs.* IgG + BM-MSCs, both biomarkers p < 0.05; Fig. 2c, d). Depletion of the Treg pool by PC61 administration significantly increased CK and LDH in the serum compared with those receiving IgG alone (IgG *vs.* PC61, both biomarkers p < 0.05; Table 1 and Fig. 2c, d), and this was suppressed by co-administration of BM-MSCs to replenish the Treg pool depleted by PC61 (PC61 *vs.* PC61 + BM-MSCs, both p < 0.01; Table 1 and Fig. 2c, d). No significant differences were found in the serum levels of CK and LDH between mice receiving PC61 + BM-MSCs and those receiving isotype and saline control (IgG *vs.* PC61 + BM-MSCs, both biomarkers p > 0.05; Table 1 and Fig. 2c, d).

### Myocardial injury induced by ischemia–reperfusion was ameliorated by administration of BM-MSCs

**Fig. 3:**
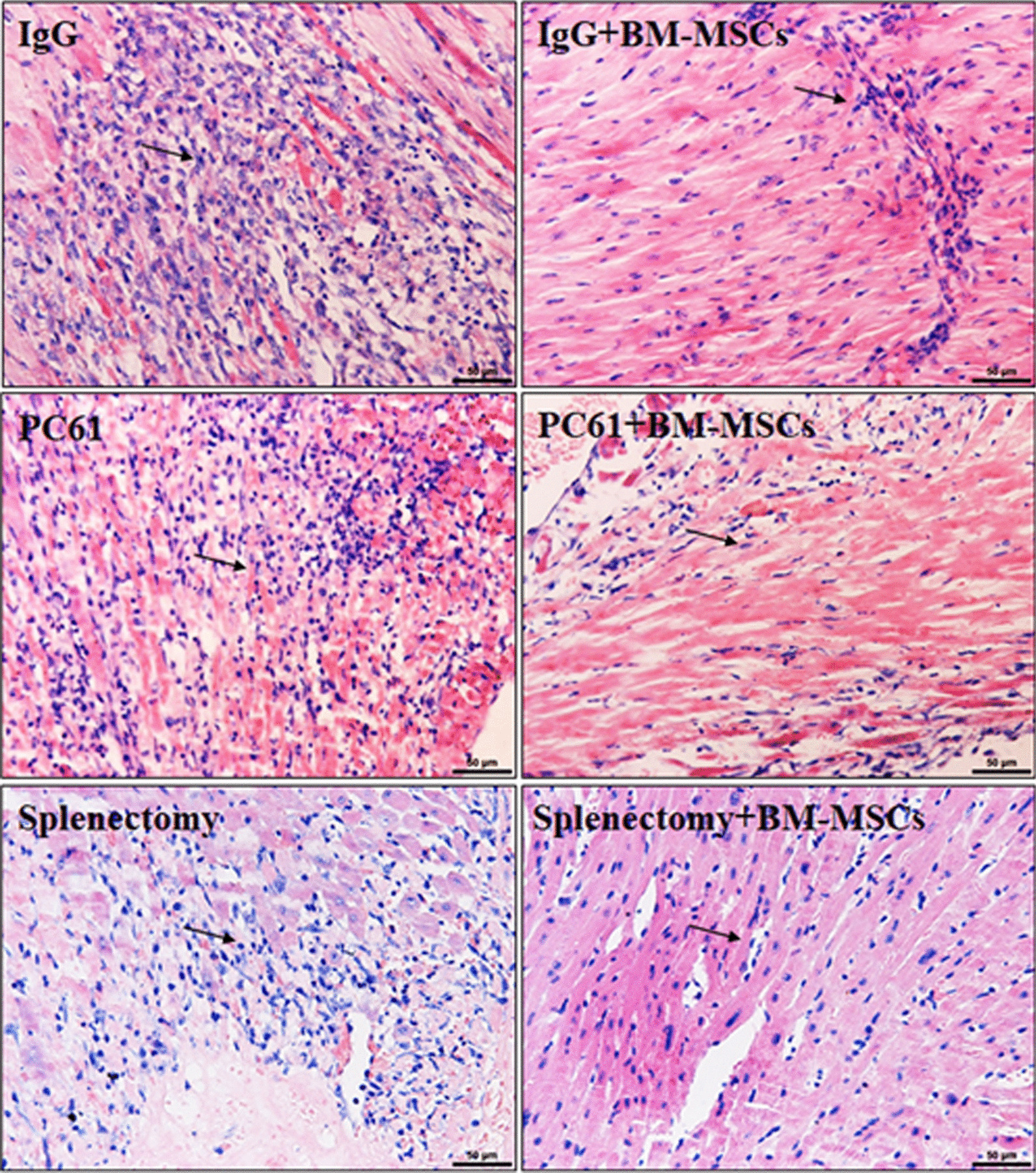
Cardiac infiltrating cells and cardiomyocyte disruption were reduced after BM-MSCs treatment in MIRI mice. Images (200×) of HE-stained myocardium sections of representative MIRI mice treated with IgG, IgG + BM-MSCs, PC61, PC61 + BM-MSCs, splenectomy, and splenectomy + BM-MSCs are shown. The hearts were collected 72 h after surgically-induced MIRI. Arrows indicate the mononuclear cells. The scalebar shown in the lower right corner of each image represents 50 μm. BM-MSCs, bone marrow-derived mesenchymal stem cells; MIRI, myocardial ischemia–reperfusion injury

H&E staining of heart tissue sections revealed that cardiomyocytes had been replaced by loose fibro-connective tissues after the induction of MIRI, accompanied by fibroblast proliferation and inflammatory cell infiltration (Fig. 3, upper left panel). The administration of BM-MSCs assisted with retaining the structure and distribution of cardiomyocytes, with limited edema and inflammatory foci (Fig. 3, upper right panel). The histologic findings of heart tissue treated with PC61 alone showed clearance of necrotic cardiomyocytes and decreased staining of the endocardial infarct, which was replaced by loose fibro-connective tissues, edema, and numerous infiltrating inflammatory cells (Fig. 3, middle left panel). In contrast, the heart tissue sections of mice treated with PC61 + BM-MSCs exhibited limited infiltration of mononuclear cells and fibrogenesis.

### BM-MSCs reduced apoptotic events and area of MI in the myocardium of MIRI mice

**Fig. 4:**
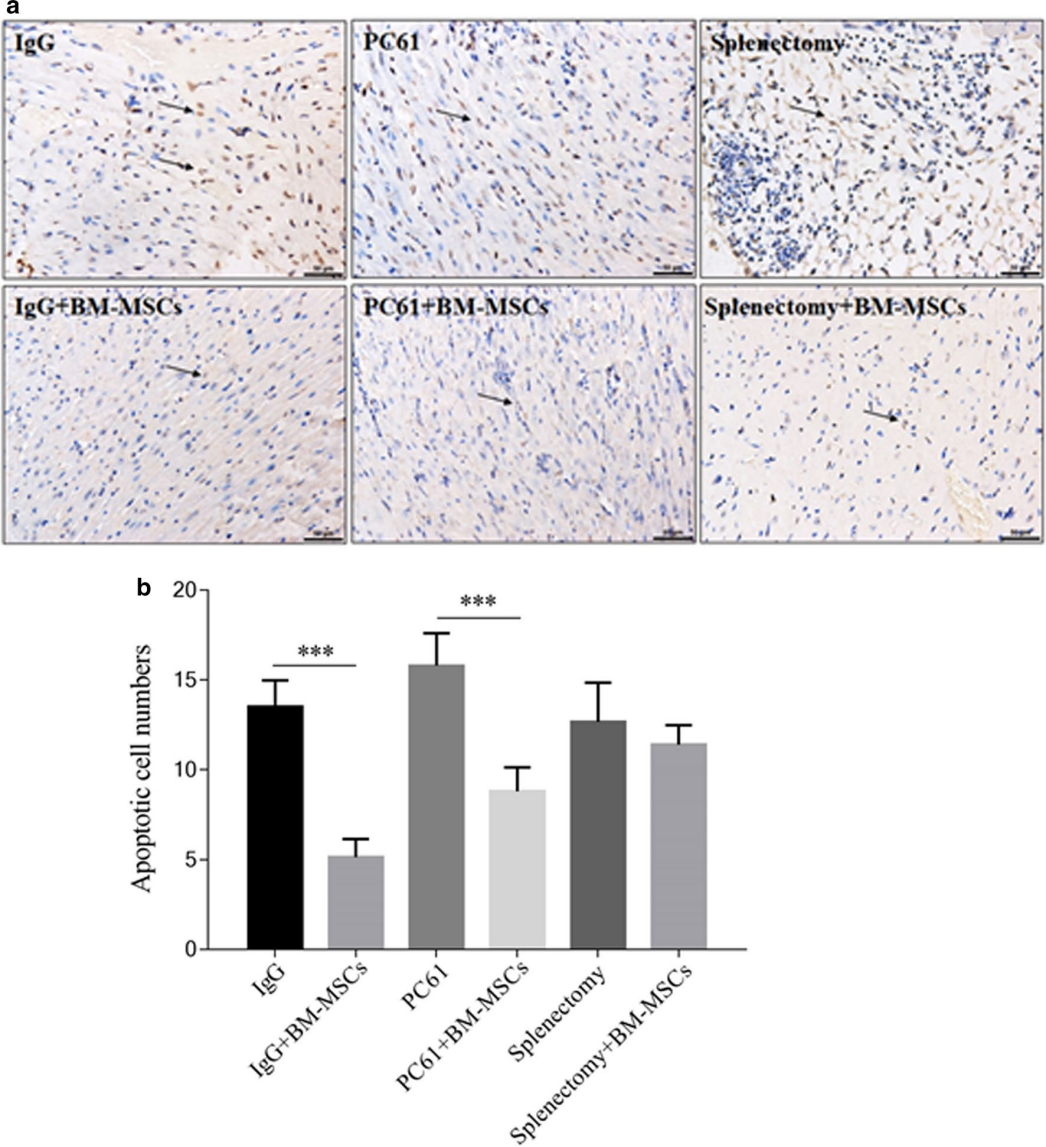
BM-MSCs treatment decreased the cardiac apoptotic cell numbers after MIRI. Representative images (200×) of myocardial TUNEL staining of MIRI mice treated with IgG, IgG + BM-MSCs, PC61, PC61 + BM-MSCs, splenectomy, and splenectomy + BM-MSCs are shown (**a**). The hearts were collected 72 h after surgically-induced MIRI. Arrows indicate the apoptotic cells. The scalebars shown in the lower right corner of each panel represent 50 μm. From the images, the number of apoptotic cells were counted and expressed in mean value ± standard deviation of each indicated group (n = 5) (**b**). BM-MSCs, bone marrow-derived mesenchymal stem cells; MIRI, myocardial ischemia–reperfusion injury

**Fig. 5:**
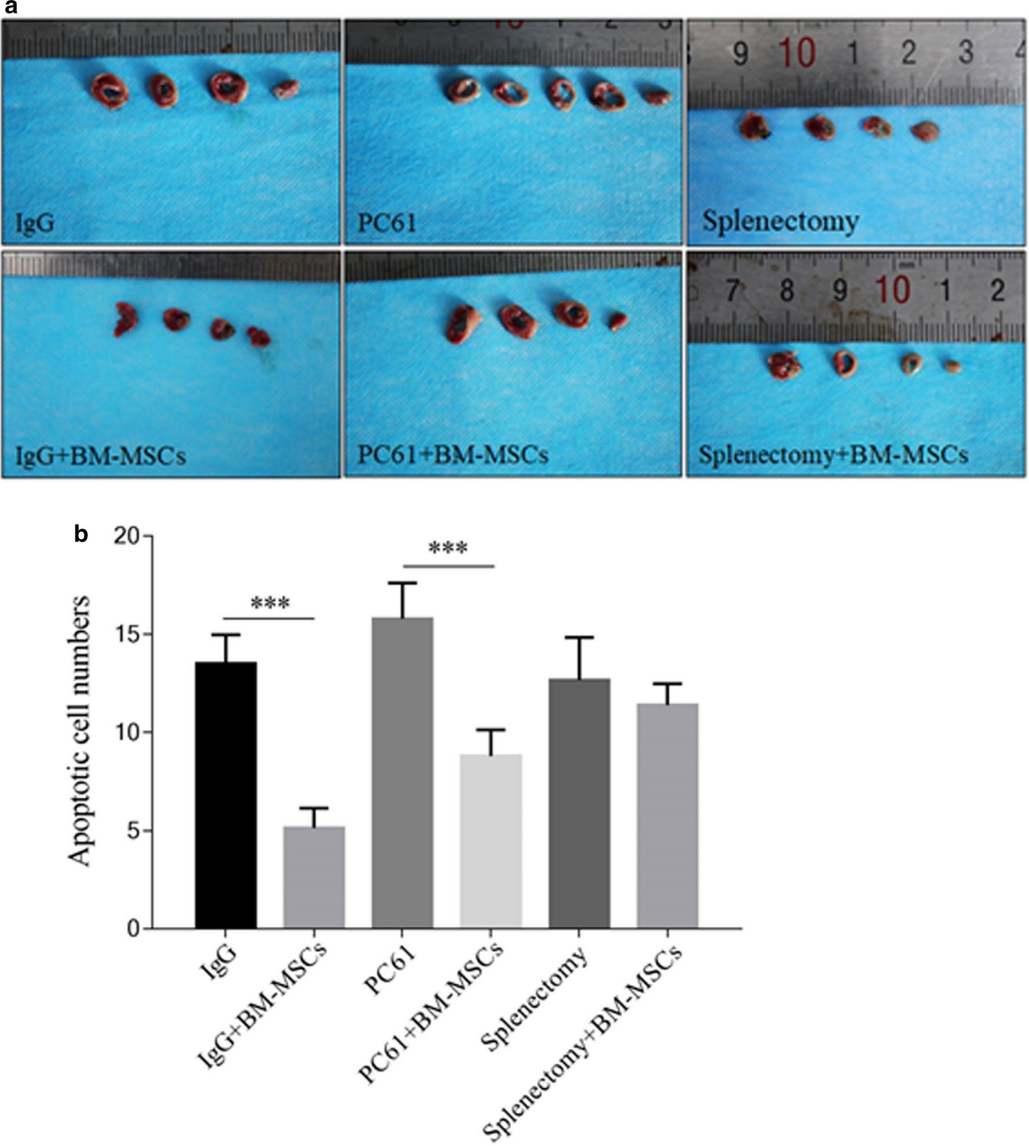
MI area was attenuated by BM-MSCs treatment after MIRI induction. Photographs of TTC stained myocardium tissues of representative MIRI mice treated with IgG, IgG + BM-MSCs, PC61, PC61 + BM-MSCs, splenectomy, and splenectomy + BM-MSCs are shown (**a**). The hearts were collected 72 h after surgically-induced MIRI. From the micrographs, the area of MI (stained grey by TTC) was calculated and expressed in mean value ± standard deviation of each indicated group (n = 5) (**b**). BM-MSCs, bone marrow-derived mesenchymal stem cells; MI, myocardial infarction; MIRI, myocardial ischemia–reperfusion injury; TTC, 2,3,5-triphenyltetrazolium

The number of apoptotic cells in the myocardium and the area of MI at 72 h post-MIRI was significantly reduced by administration of BM-MSCs (IgG *vs* IgG + BM-MSCs, both p < 0.05; Table 2, Figs. 4, 5). Similarly, the addition of BM-MSCs to replenish the Treg pool depleted by PC61 ameliorated the myocardial injury observed after induction of MIRI (PC61 *vs* PC61 + BM-MSCs, both p < 0.05; Table 2, Figs. 4, 5). Of specific interest, the extent of myocardial injury and cellular apoptosis was similar between those treated with PC61 + BM-MSCs and IgG alone (both p > 0.05, Figs. 4, 5).

**Table 2:** The myocardium infarction area in each group after myocardial ischemia–reperfusion injury

Group	Infarction area $(\overline{x} \pm {\text{SD}},\% )$
IgG	31.13 ± 6.82
IgG + BM-MSCs	20.15 ± 1.22
PC61	54.79 ± 7.65
PC61 + BM-MSCs	39.68 ± 4.12
Splenectomy	57.30 ± 11,091
Splenectomy + BM-MSCs	52.53 ± 8.55

### Splenocytes supported the immunomodulatory, Treg-promoting, and anti-apoptotic effects of BM-MSCs in MIRI mice

Splenectomy diminished the level of cardiac Tregs and immunomodulatory cytokines induced by BM-MSCs (splenectomy *vs.* splenectomy + BM-MSCs, all p > 0.05; Table 1 and Figs. 1, 2). No significant differences were observed in MIRI biomarkers, apoptotic cell numbers, myocardium infarction area, and the histological findings between splenectomized MIRI mice receiving BM-MSCs and those receiving saline (all p > 0.05, Figs. 2, 3, 4, 5). The Treg population was significantly increased after co-culturing splenocytes with BM-MSCs ex vivo, and the production of IL-10 and TGF-β1 was similarly enhanced (all p < 0.05, Additional file 1: Figure S1).

## Discussion

Results of the present study illustrate the therapeutic capacity and the potential working mechanism of BM-MSCs transfusions in an experimentally-induced MIRI preclinical mouse model. BM-MSCs facilitated the expansion of Tregs in the heart and spleen of MIRI mice, which was accompanied by a concurrent increase in immunomodulatory cytokines IL-10 and TGF-β1. The extent of injury induced by MI was attenuated by BM-MSCs and Tregs, and this phenomenon was reduced by the depletion of Tregs. The therapeutic effects of BM-MSCs and the participation of Tregs was supported by the histopathological observations and serum biomarkers (i.e., CK and LDH). Meanwhile, these results have demonstrated that the spleen is the pivotal organ by which the transfused BM-MSCs can nurture Tregs, promote immunomodulation, and hence, alleviate MIRI.

The therapeutic and protective effects of BM-MSCs treatment for ischemia–reperfusion injury have been investigated in several organs (17–20). In particular, the role and potential of BM-MSCs in ameliorating MIRI has been previously demonstrated (21–23). It has been shown that BM-MSCs differentiate into cardiomyocytes under appropriate ex vivo culture conditions (22, 23). Earlier animal studies reported that BM-MSCs transfusions promoted cardiomyocyte proliferation and angiogenesis (21). In doing so, BM-MSCs prevented myocardial fibrosis and remodeling after ischemia, leading to improved cardiac function (21). The phase II and III clinical trials have examined the efficacy of BM-MSCs treatment in acute MI patients, and demonstrated an increased left ventricular ejection fraction, myocardial reperfusion, and ventricular remodeling (10).

Studies have shown that BM-MSCs induce the in vitro differentiation of Tregs through secretion of IL-10 and TGF-β1 (24, 25), and facilitate the inhibition of inflammation and associated tissue injury (24). The most studied Treg population in the context of MIRI, are CD4^+^Fopx3^+^ Tregs (26). Previous studies have demonstrated the significance of CD25 in Treg function (27), and the depletion of Tregs was achieved by anti-CD25 PC61 antibody without affecting the function of other immune cells (28). The present study has successfully demonstrated the pivotal role of Tregs in BM-MSCs treatment of MIRI through the observation of increased numbers of Tregs after BM-MSCs administration and depletion of the Treg pool by adopting PC61 antibody. While promotion of the Treg population and immunomodulatory cytokines (IL-10 and TGF-β1) was observed concurrently with the amelioration of apoptosis and tissue injury in the myocardium, the direct cellular and molecular pathway of immunomodulation remains to be addressed. Of specific note, the depletion of Tregs by PC61 alone did not translate into a significantly reduced immunomodulatory cytokine in the serum, suggesting that other cell types may be involved, at least within the first 72 h after MIRI.

The inflammatory response induced by MIRI involves neutrophil infiltration and M1 polarization of macrophages, and as a result, exhibits a high expression of proinflammatory cytokines (e.g., IL-1β, IL-6, and TNF-α) and suppressed immunomodulatory factors such as IL-10 and TGF-β1 (29). Tregs modulate mature T cell responses through the inhibition of immune activation and establishment of immune tolerance, leading to a dampened inflammatory response (30). In MIRI, Tregs are able to revert M1 polarization to M2, enhance collagen production and angiogenesis, induce anti-inflammatory cytokines, improve ventricular remodeling, and facilitate recovery of the ischemic myocardium (31, 32). In addition, Tregs have been shown to improve post-MIRI cardiac functions via suppressing the activation of matrix metalloproteinase 2 (MMP-2) (26). Through inhibiting the MMP-2 pathway, Tregs were shown to attenuate myocardium cellular apoptosis and extracellular matrix remodeling (33). The findings from that MIRI preclinical study illustrate the importance of IL-10 and TGF-β1 in Tregs and BM-MSCs-attenuated myocardial injury. The participation of the MMP-2 pathway and its anti-apoptotic role are currently under investigation and deserve to be the focus of future studies.

Parekkadan et al. (2011) observed previously that co-culturing BM-MSCs cells with splenocytes resulted in increased numbers of Tregs in the presence of IL-2 (16). Additionally, transplantation of the splenocytes into a murine colitis model prolonged animal survival and enhanced the Treg population in the mesenteric lymph nodes, while these effects were eliminated by splenectomy (16). Corroborating the enhanced Treg pool in secondary lymphoid organs, the engraftment of exogenous BM-MSCs in the inguinal, mesenteric, and pancreatic lymph nodes was previously found to occur coincidentally with the therapeutic activities of BM-MSCs in streptozotocin-induced diabetic mice (34). Promotion of BM-MSCs’ nurturing of the spleen through splenocyte activity augmented the anti-inflammatory effects of BM-MSCs in mice with colitis induced by dextran sulfate sodium (35). The present study expanded these findings and further demonstrated the importance of the spleen as a Treg nurturing site post-MIRI as illustrated by the findings of splenectomized mice in the present experiments. The cardiac Treg pool induced by the administration of BM-MSCs after MIRI induction was abrogated in the splenectomized mice. Thus, further studies should be conducted to investigate the molecular mechanisms of BM-MSCs-promoted Treg activation in the spleen, which ameliorates MIRI-induced inflammatory and injury.

Ischemic injury is typically accompanied by a deeper grade of myocardial tissue hypoxia, generating increased the production of reactive oxygen species (ROS), which in turn activates maladaptive signaling pathways, leading to myocardial cell death and maladaptive cardiac remodelling (36). Carbonic anhydrases (CAs) are a family of ubiquitous metalloenzymes that are responsible for the rapid conversion of carbon dioxide to bicarbonate and protons and thus are involved in a variety of physiological and pathological processes that involve pH regulation, CO2 and HCO3 transport, ion transport, and biosynthetic reactions (37). The CA inhibitor ethoxyzolamide prevents rat cardiomyocyte hypertrophy in vitro and reverses it once it is established (38). Elevated CA-II expression has been detected in rats with spontaneous hypertension and heart failure (39). CA-I myocardial expression regulates with capillary density and endothelial cell death (40), which correlated with myocardial ischemia–reperfusion injury (41). Recent report revealed that the depletion of CA-I increases the activation of Treg cell (42). Taken together, these evidences also suggest that a mechanism similar to that underlying CA-II increase in myocytes may also explain CA-I increase in endothelial cells in myocardial ischemia–reperfusion injury. Certainly, this hypothesis remains highly speculative, and it remains to be properly tested in future studies.

Otherwise, to explore the CD25 + Foxp3 + Treg cells function in myocardial ischemia–reperfusion injury, which drive from BM-MSCs, we defined the significantly effects of adoptive transfer of BM-MSCs. In post-MIRI remodeling, multiple immune cell types infiltrate the infracted heart, including neutrophils, infiltrating pro-inflammatory monocytes (43). Study reported that CD4 + T cells (including Foxp3 + Tregs) infiltrated the ischemia injury heart and promote wound healing via favorably monocytes and/or macrophage trafficking and differentiation (44). Additional, splenocytes obviously mediated a broad array of pro-inflammatory factors. The current results were demonstrated a robust tissue-injurious cardiosplenic axis in ischemic heart reperfusion injury, which consistent with pervious works, However, we did not transfer splenocytes into the MIRI recipient mice to explore the influence of CD25 + Foxp3 + Treg cells after splenectomized. Still, we did not evaluate the myocardial function after transfer BM-MSCs in MIRI recipient mice. Thus, the clear mechanisms need to investigate in the future. Clinical application may require a large dose of Treg cells, and one challenge is hard to expand and distinguish human Treg cells. Results from Taams et al. suggested that CD4 + CD25 + Foxp3 + Treg cells can polarize macrophage transition in the inflammation site under tissue repairing (45). CD127, an IL-7 has been reported could be as an appropriate marker for natural Treg cell isolation for clinical testing (46). Study demonstrated that total Treg cell number of human body is around 1.3 × 10^10^, while the circulation Treg cells number is around 0.2 × 10^9^ without expansion (47). These studies indicated that the limitation for the application of human-obtained Tregs. Despite these limitation, BM-MSCs exhibited the advantage in treatment of ischemia–reperfusion injury in comparison of Treg cells and provide another therapeutic strategy in clinical application.

## Conclusion

In conclusion, BM-MSCs ameliorate MIRI-induced inflammatory and cellular apoptosis in the myocardium via the induction of Tregs and upregulation of IL-10 and TGF-β1. The spleen is the key organ facilitating BM-MSCs-directed promotion of Tregs. However, the precise molecular and cellular components involved in the immunomodulatory mechanism of BM-MSCs warrant more in-depth investigation, especially in the context of MIRI.

## Supplementary Information

**Additional file 1.** **Supplemental Fig 1.** BM-MSCs cocultured with splenocytes induced the Treg population and increased the levels of IL-10 and TGF-β1. Splenocyte (5× 106 cells) were freshly isolated from mice and cocultured with BM-MSCs (1×105 cells) for 72 h. Representative flow cytometry scatter plots (A) and qualification results (B) confirmed CD25^+^Foxp3^+^ Tregs from splenocytes and splenocytes+BM-MSCs. The cocultured supernatant were examined IL-10 and TGF-β1 by ELISA. SEM ± SD, **p < 0.01 and ***p < 0.001.

## Data Availability

All data generated or analyzed during this study are included in this published article.

## Abbreviations

MI: Myocardial infarction
MIRI: Myocardial ischemia–reperfusion injury
BM-MSCs: Bone marrow-derived mesenchymal stem cells
Tregs: Regulatory T cells
IgG: Immunoglobulin G
IL-10: Interleukin 10
TGF-β1: Transforming growth factor β1
CK: Creatinine kinase
LDH: Lactate dehydrogenase
LAD: Left anterior ascending coronary artery
PBS: Phosphate buffered saline
RBC: Red blood cells
H&E: Hematoxylin and eosin
MMP2: Matrix metalloproteinase 2
