# Supplementary material for: Bone marrow-derived mesenchymal stem cells attenuate myocardial ischemia–reperfusion injury via upregulation of splenic regulatory T cells
Source: BMC Cardiovasc Disord. 2021 Apr 27;21:215. doi: 10.1186/s12872-021-02007-4 (PMC8080373; doi:10.1186/s12872-021-02007-4)
Supplement: Supplementary file 1 — Additional file 1. Supplemental Fig 1. BM-MSCs cocultured with splenocytes induced the Treg population and increased the levels of IL-10 and TGF-β1. Splenocyte (5× 10^6^ cells) were freshly isolated from mice and cocultured with BM-MSCs (1×10^5^ cells) for 72 h. Representative flow cytometry scatter plots (A) and qualification results (B) confirmed CD25+Foxp3+ Tregs from splenocytes and splenocytes+BM-MSCs. The cocultured supernatant were examined IL-10 and TGF-β1 by ELISA. SEM ± SD, **p < 0.01 and ***p < 0.001. [file 12872_2021_2007_MOESM1_ESM.docx]

**Bone Marrow-Derived Mesenchymal Stem Cells Attenuate Myocardial Ischemia-Reperfusion Injury Via Upregulation of Splenic Regulatory T Cells**

**Running Title:** Tregs alleviate myocardial infarction.

Ling-Xiao Pang, MD^1^; Wen-we Cai, PhD^1^; Qian Li, PhD^1^, Heng-Ji Li, PhD^1^; Min Fei, PhD^2^; Yong-Shen Yuan, MD^1^, Bin Sheng, MD^1^; Ke Zhang, MD^1^; Rong-Cheng An, MD^1^, Ying-Wei Ou, MD^1^; Wen-Jie Zeng, MD^3^*

^1^ Department of Emergency, Zhejiang Provincial People’s Hospital, People’s Hospital of Hangzhou Medical College, Hangzhou 310014, China

^2^ Department of health management center, Zhejiang Provincial People’s Hospital, People’s Hospital of Hangzhou Medical College, Hangzhou 310014, China

^3^ Department of Gynecology, Zhejiang Provincial People’s Hospital, Hangzhou Medical College, People’s Hospital, Hangzhou 310014, China

*Correspondence:

Wen-Jie Zeng, MD

Department of Gynecology, Zhejiang Provincial People’s Hospital, ShangTang Road 158, 310014 Hangzhou, China.

Tel: +86-0571-85893634.

E-mail: DRpanglingxiao@163.com

**Additional file 1: Figure S1**

**
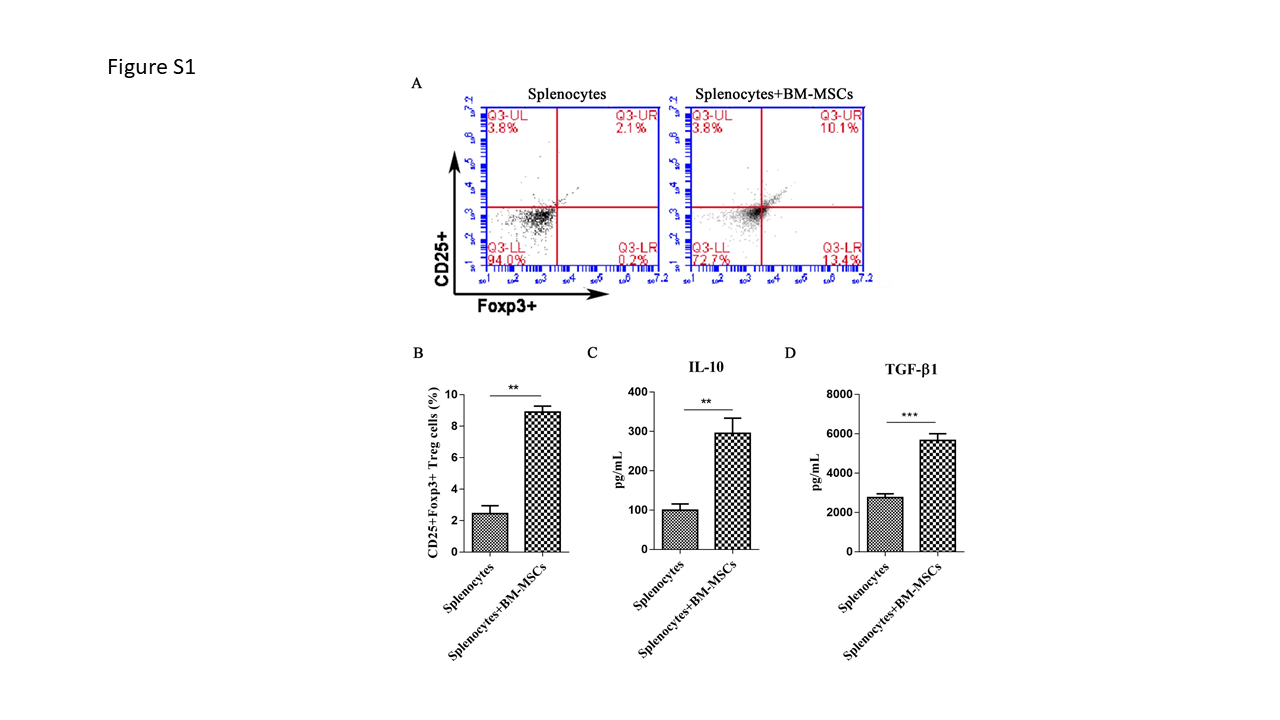
**
